# The innate immune factor RPRD2/REAF and its role in the Lv2 restriction of HIV

**DOI:** 10.1128/mbio.02572-21

**Published:** 2023-10-26

**Authors:** Kathryn A. Jackson-Jones, Áine McKnight, Richard D. Sloan

**Affiliations:** 1 Centre for Inflammation Research, Institute of Regeneration and Repair, The University of Edinburgh, Edinburgh, United Kingdom; 2 Division of Infectious Diseases & Department of Microbiology-Immunology, Northwestern University Feinberg School of Medicine, Chicago, Illinois, USA; 3 Blizard Institute, Barts and The London School of Medicine and Dentistry, Queen Mary University of London, London, United Kingdom; 4 ZJU-UoE Institute, Zhejiang University, Haining, China; Ohio State University, Columbus, Ohio, USA; University of California, Berkeley, California, USA

**Keywords:** human immunodeficiency virus, retroviruses, innate immunity, transcription, reverse transcription, lentiviral restriction

## Abstract

Intracellular innate immunity involves co-evolved antiviral restriction factors that specifically inhibit infecting viruses. Studying these restrictions has increased our understanding of viral replication, host-pathogen interactions, and pathogenesis, and represent potential targets for novel antiviral therapies. Lentiviral restriction 2 (Lv2) was identified as an unmapped early-phase restriction of HIV-2 and later shown to also restrict HIV-1 and simian immunodeficiency virus. The viral determinants of Lv2 susceptibility have been mapped to the envelope and capsid proteins in both HIV-1 and HIV-2, and also viral protein R (Vpr) in HIV-1, and appears dependent on cellular entry mechanism. A genome-wide screen identified several likely contributing host factors including members of the polymerase-associated factor 1 (PAF1) and human silencing hub (HUSH) complexes, and the newly characterized regulation of nuclear pre-mRNA domain containing 2 (RPRD2). Subsequently, RPRD2 (or RNA-associated early-stage antiviral factor) has been shown to be upregulated upon T cell activation, is highly expressed in myeloid cells, binds viral reverse transcripts, and potently restricts HIV-1 infection. RPRD2 is also bound by HIV-1 Vpr and targeted for degradation by the proteasome upon reverse transcription, suggesting RPRD2 impedes reverse transcription and Vpr targeting overcomes this block. RPRD2 is mainly localized to the nucleus and binds RNA, DNA, and DNA:RNA hybrids. More recently, RPRD2 has been shown to negatively regulate genome-wide transcription and interact with the HUSH and PAF1 complexes which repress HIV transcription and are implicated in maintenance of HIV latency. In this review, we examine Lv2 restriction and the antiviral role of RPRD2 and consider potential mechanism(s) of action.

## INTRODUCTION

While the majority of virological research uses laboratory-adapted viruses, increasingly, it has been acknowledged how distinct these adapted strains are from the primary virus they are being used to model (1–3). For example, laboratory- adapted viruses can often overcome host restriction factors that inhibit primary virus. These restriction factors have been discovered either by defining the genetic determi­ nants of unmapped restrictions using phenotypic mapping or through unbiased screens that identify novel restriction factors. A multitude of host intracellular innate immune mechanisms that target retroviral infection have now been described at all stages of the HIV replication cycle (reviews in references 4, 5; examples in references 6–15). The first unmapped lentiviral restriction was identified in 1956, although the viral agent was at that time unknown. Lentiviral restriction 1 (Lv1) (reviewed in reference 16) as it was named in 2002 was described as a block to lentivirus infection occurring prior to or concurrently with reverse transcription (17–20) and in 2004 was mapped to TRIM5α (20, 21).

In 2001, McKnight et al. published a comparison of a primary isolate of HIV-2 (prCBL-23) and a laboratory-adapted isolate (CBL-23) cultured from the same individual. Strikingly, they found that while laboratory-adapted CBL-23 could productively infect actively dividing CD4-expressing HeLa and osteosarcoma cell lines, as well as primary macrophages, the primary virus was restricted (22). This novel host restriction that the laboratory-adapted virus had apparently overcome was named lentiviral restriction 2 (Lv2) in 2004 (23).

**Fig 1:**
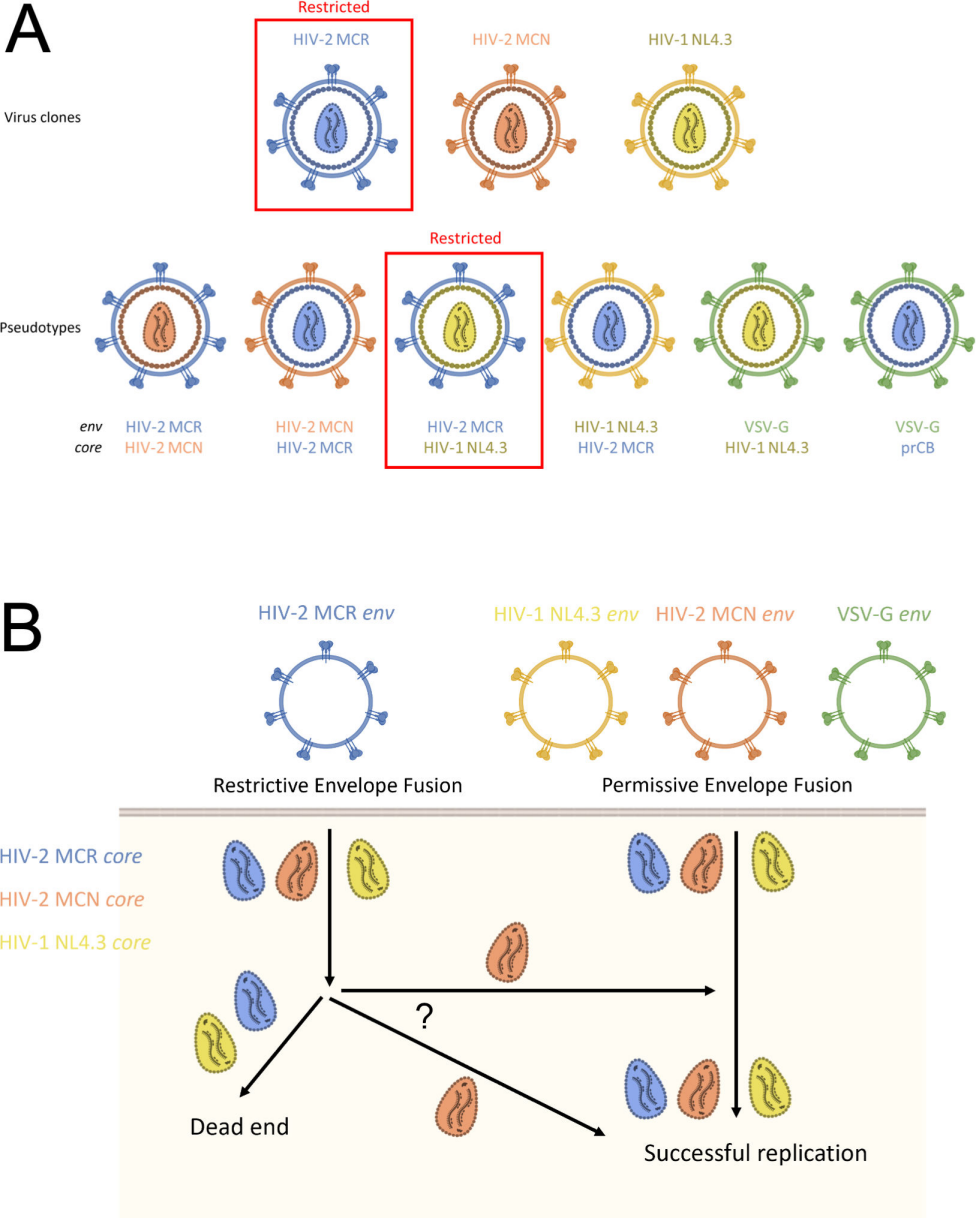
Pseudotyping experiments reveal that Lv2-mediated restriction affects different route of entry mechanisms of HIV. (A) Schematic of the three HIV viral clones and six different pseudotypes tested for Lv2 susceptibility by either McKnight et al. and/or Schmitz et al. (23, 24). For pseudotypes, the viral source of both the envelope and core is shown. Virions restricted by Lv2 are indicated by a red box. MCR, molecular clone restricted; MCN, molecular clone non-restricted; VSV-G, vesicular stomatitis virus glycoprotein; prCB, primary patient isolate of HIV-2. (B) Model to explain the results from infection experiments using viral clones and pseudotypes shown in panel A. Several routes of entry are likely with potential re-routing between them. Adapted from reference 23.

To investigate whether viral entry to the cell was affected by Lv2, the transfection reagent 1,2-dioleoyl-3-trimethylammonium propane (DOTAP), which aids fusion with the cell membrane, was utilized. DOTAP treatment enhanced infection by the primary isolate prCBL-23 to levels comparable to those with CBL-23, indicating that Lv2-mediated restriction affects viral entry (22). However, prCBL-23 efficiently induced fusion with cell membranes (22), so the entry deficiency was not due to a defective prCBL-23 envelope. Creating a pseudotype by adding an envelope glycoprotein G from the vesicular stomatitis virus (VSV-G) to prCBL-23 also rescued infection (Fig. 1A) (22). HIV virions bearing VSV-G enter cells through an endocytic pathway, since this is the normal route of VSV infection (24). Therefore, it was suggested that the VSV-G envelope bypassed Lv2 restriction by utilizing the endocytic pathway and that the pre-integration complexes of the restricted primary virus were trapped in a restrictive cellular location, preventing subsequent steps in the replication cycle (Fig. 1B). Since Lv1 restriction had been characterized as independent of viral route of entry (25), the evidence that Lv2-mediated restriction was overcome with VSV-G pseudotyped HIV-2 isolates (22) confirmed that it was a distinct restrictive mechanism from Lv1. This was some of the first evidence that the delivery of the virion core into an appropriate cellular compartment is an important step in the HIV replication cycle. One suggested mechanism was that following membrane fusion, the unrestricted core could connect with the appropriate cytoskeletal element to facilitate transport to the nucleus and that the restricted, primary isolate prCBL-23 was unable to do this (22).

Using endpoint PCR, complete DNA reverse transcription products were detected 24 hours after infection by either prCBL-23 virus or CBL-23 virus in primary macrophages and primary blood mononuclear cells (PBMCs), showing that Lv2 does not completely inhibit reverse transcription. However, as quantitative PCR had not yet been developed, quantification was qualitative. At later time points, there was a reduction to undetectable levels of viral DNA transcripts in the prCBL-23-infected primary macrophages suggesting that transcripts were being degraded or reverse transcription inhibited.

The discovery and early characterization of Lv2 restriction was part of a growing realization that host cells have multiple mechanisms to restrict incoming viruses. At a similar point, other restrictions were being described including apolipoprotein B mRNA editing enzyme catalytic subunit 3 (APOBEC3) (26) and TRIM (27) family proteins. Three further unmapped restrictions have since been described termed Lv3 (28), Lv4 (29), and Lv5 (30). The identification of Lv2 restriction also highlights the stark differences between a laboratory-adapted virus and primary isolates, and the need to consider these differences when studying viral mechanisms. Many questions remained about Lv2 including how a restriction that manifested post-entry could be overcome by changing the route of the viral capsid delivery. Indeed, it was not known if the Lv2 phenotype was a single restriction or actually a cumulation of multiple restrictions. To characterize the Lv2 restriction further, mapping the cellular phenotype to viral genetic determinants was needed.

### Lv2-mediated restriction is opposed by both HIV capsid and envelope and is dependent on cellular entry mechanism

To further characterize Lv2 and to define its link to cellular entry, Schmitz et al. created molecular clones of the primary restricted HIV-2 prCBL-23 virus which they named MCR (molecular clone restricted), and the unrestricted laboratory-adapted HIV-2 CBL-23 virus which they named MCN (molecular clone non-restricted) (23). Sequence analysis comparing Lv2-restricted MCR to Lv2 non-restricted MCN identified amino acid altering differences in many genes but gene swapping experiments allowed the viral determinants of the Lv2 restriction to be mapped to the *gag* and *env* genes (23). The restricted or unrestricted phenotype was only partially conferred if either *gag* or *env* alone was swapped between molecular clones. However, the combination of *gag* and *env* resulted in an almost complete exchange of phenotypes (23). Site-directed mutagenesis further identified a single amino acid at position 207 of the Gag polyprotein, within the capsid protein (position 73 of capsid) to be responsible for the gag restriction (23). It was therefore concluded that both *env* and *gag* are responsible for the Lv2 restriction; however, since no single determinant was identified, the Lv2 phenotype may actually be composed of more than one restriction.

Pseudotype experiments using laboratory-adapted HIV-1 NL4.3 further indicated that the route of entry is also important for the infection of cells by HIV-1 (Fig. 1A) (23). The HIV-1 molecular clone NL4.3 was not restricted in an Lv2-like manner (23). However, the NL4.3 core was restricted if supplied with an Lv2-restricted MCR envelope, but not with VSV-G, showing that an unrestricted core is restricted if fused into the cell by restrictive envelopes. The authors hypothesized that an unrestricted envelope may also be able to rescue restricted cores. Indeed, the NL4.3 envelope could rescue the Lv2-restricted MCR core (23) (Fig. 1A). Since pseudotyping with the HIV-2 Lv2 restricted MCR envelope could render the NL4.3 core to be susceptible, this showed that Lv2 is a generalized restriction active against both HIV-2 and HIV-1. Furthermore, while cellular fusion of HIV-1 and HIV-2 is pH independent (31, 32), inhibition of endosomal acidification partially rescued the restriction of MCR, but had no effect on the infectivity of MCN (33). Therefore, the envelope-mediated restriction could be explained by alternative routes of entry into the cell. This led to the hypothesis that viral fusion into the cell can occur by at least two routes (33), including a restrictive endocytic route (34), where the viral core is delivered to a compartment where Lv2 is operative, and infection is therefore inhibited (Fig. 1B), and a permissive route at the plasma membrane. Interestingly, the pseudotyping experiments taken together suggest that an unrestrictive core (e.g., the MCN core) delivered via the restricted route (e.g., MCR envelope) can avoid Lv2-mediated restriction. It is likely that multiple distinct post-entry pathways exist with different outcomes for different capsids (Fig. 1B). However, the NL4.3 core delivered via the restricted route was still restricted, an observation that is not yet understood but suggests that distinct HIV cores could differently be targeted by or evade host restriction factors. Nevertheless, unrestricted envelope and capsid were both necessary to ensure an entry route that allows post-entry events in viral replication to proceed efficiently without being affected by Lv2 restriction.

Further insight came from Reuter et al., who showed that the single point mutation in the envelope protein of Lv2-restricted MCR resulted in higher affinity CD4 binding compared to unrestricted MCN, which enhanced endocytosis resulting in a less efficient fusion into the cytosol (35). The authors suggested that this entry defect largely accounted for the reduced infectivity of MCR; however, it does not explain why an MCN core is unrestricted even when pseudotyped with an MCR envelope. HIV can fuse with cells either at the plasma membrane or via an endocytic route. It is contentious whether the endocytic route is pH dependent and whether this fusion pathway leads to productive infection (36, 37). Prevention of endosome formation and prevention of endosome acidification enhanced infectivity of Lv2-restricted MCR (35), indicating that a degradative lysosomal pathway could be the cause of the reduced cytosolic entry. However, a remaining restriction to infection (19-fold) was preserved when endosomal acidification was prevented (35). Therefore, the entry defect does not completely account for Lv2-mediated restriction to infection, which is also dependent on the capsid.

These data were corroborated by Marchant et al. who used compounds that affect endocytic pathways or lipid rafts to interrogate the mechanism of Lv2-restricted MCR cellular entry (33). They showed that a restricted virus could be rescued from Lv2 if a lipid raft-dependent, pH-independent endocytic pathway was inhibited. Furthermore, entry of Lv2-restricted MCR into HeLa/CD4 cells expressing a tail-less CD4 receptor, that localizes outside lipid rafts, was unrestricted. Building on earlier experiments, they also showed that 5 of 10 tested HIV-2 isolates and 5 of 12 tested HIV-1 isolates were susceptible to Lv2, confirming that this restriction is broad-acting (33). It was consistently noted that MCR infection was restricted in some cell lines but absent in others (23), further indicating that cellular components are also responsible for the Lv2 restriction.

Although various virological and pharmacological infection assays have been used to investigate Lv2, the cellular entry mechanisms of restricted and unrestricted viral particles is not fully understood, suggesting that systematic testing of these parameters would be beneficial. It is also possible that several routes of HIV entry are possible, leading to the results observed. The Lv2 phenotype therefore could be the result of two or several restriction factors with differing efficiency depending on entry pathway. Whether different entry routes lead to different infection outcomes is unknown, although some evidence for this exists; nuclear import pathway has been linked to integration and replication efficiency (38), interferon induced transmembrane protein (IFITM) sensitivity of HIV-1 strains is determined by the co-receptor usage of the viral envelope glycoproteins (39), and nuclear entry mechanism has been shown to affect the potency of the restriction factor Mx2/MxB (40). However, it is clear that both the envelope and capsid serve as viral determinants for Lv2 restriction of both HIV-1 and HIV-2. Furthermore, entry defects and viral determinants did not account for all reduced infectivity seen, pointing to a cellular component of Lv2 restriction.

### Identification of host factors mediating Lv2 restriction

Although the viral determinants of Lv2 had been mapped and characterized, the host factor(s) responsible was still unknown. At the time, however, siRNA screening methods capable of knocking out individual genes across the whole human genome were being increasingly used. Therefore, in 2011, Liu et al. performed a whole-genome siRNA screen to identify cellular restriction factors that might be responsible for Lv2 (41). One hundred and fourteen factors were identified that significantly inhibited infection of HIV-2 pseudotyped virions with the MCR envelope. The genes identified were involved in a broad spectrum of cellular processes including receptor signaling, vesicle trafficking, transcription, apoptosis, cross-nuclear membrane transport, meiosis, DNA damage repair, ubiquitination, and RNA processing (41).

Crucially, the pseudotyped virus used in the screen was only capable of a single round of infection and the readout was green fluorescent protein (GFP) signal driven by an HIV-1 promoter. Therefore, identified factors must affect the HIV replication cycle during entry, reverse transcription, integration, and/or gene expression. For example, tetherin, which acts at the late phase of the viral replication cycle and prevents viral budding, was, as expected, not identified in the screen. Consistent with entry being important for Lv2 restriction, clathrin adaptor complex 2 (AP-2) and dynamin, both involved in endocytosis, were identified as hits (41). Of the 10 highest confidence factors identified, three were components of the polymerase-associated factor 1 (PAF1) complex and two were members of, or closely associated with, the then undefined human silencing hub (HUSH) complex (41). The conserved PAF1 complex (reviews in references 42, 43) can both stimulate and inhibit RNA polymerase II via promoter-proximal pausing and has been implicated in gene transcription, chromatin remodeling, cell cycle control, DNA repair, and mRNA surveillance (42, 44, 45). PAF1 complex components are highly expressed in primary monocytes, macrophages, and T-lymphocytes (46). Knockdown of all members of the PAF1 complex as well as HUSH complex interactor Su(var)3-9, Enhancer-of-zeste and Trithorax (SET) domain bifurcated histone lysine methyltransferase 1 (SETDB1) have been found to enhance HIV-1 reverse transcription and subsequent integration of provirus, whereas overexpression of PAF1 complex proteins in host cells rendered them resistant to HIV-1 (41). The HUSH complex (reviews in references 47–49) has also since been well documented as antiviral, acting post-integration, specifically through its recruitment of SETDB1 (50) to deposit the repressive histone mark H3K9me3 on endogenous retroviruses (51, 52) and integrated HIV-1 provirus (53–56). There is no evidence that HUSH binds viral DNA; however, several studies have shown that the HUSH complex functions to repress transcription at HIV-1 DNA (54–56), although several other studies showed that HUSH had no effect on HIV-1 transcription (57–60). In J-Lat cells, which harbor a GFP reporter driven by a repressed HIV-1 long terminal repeat (LTR) promoter, both HUSH (61) and PAF1 (62) complex depletion resulted in promoter reactivation and GFP expression.

**Fig 2:**
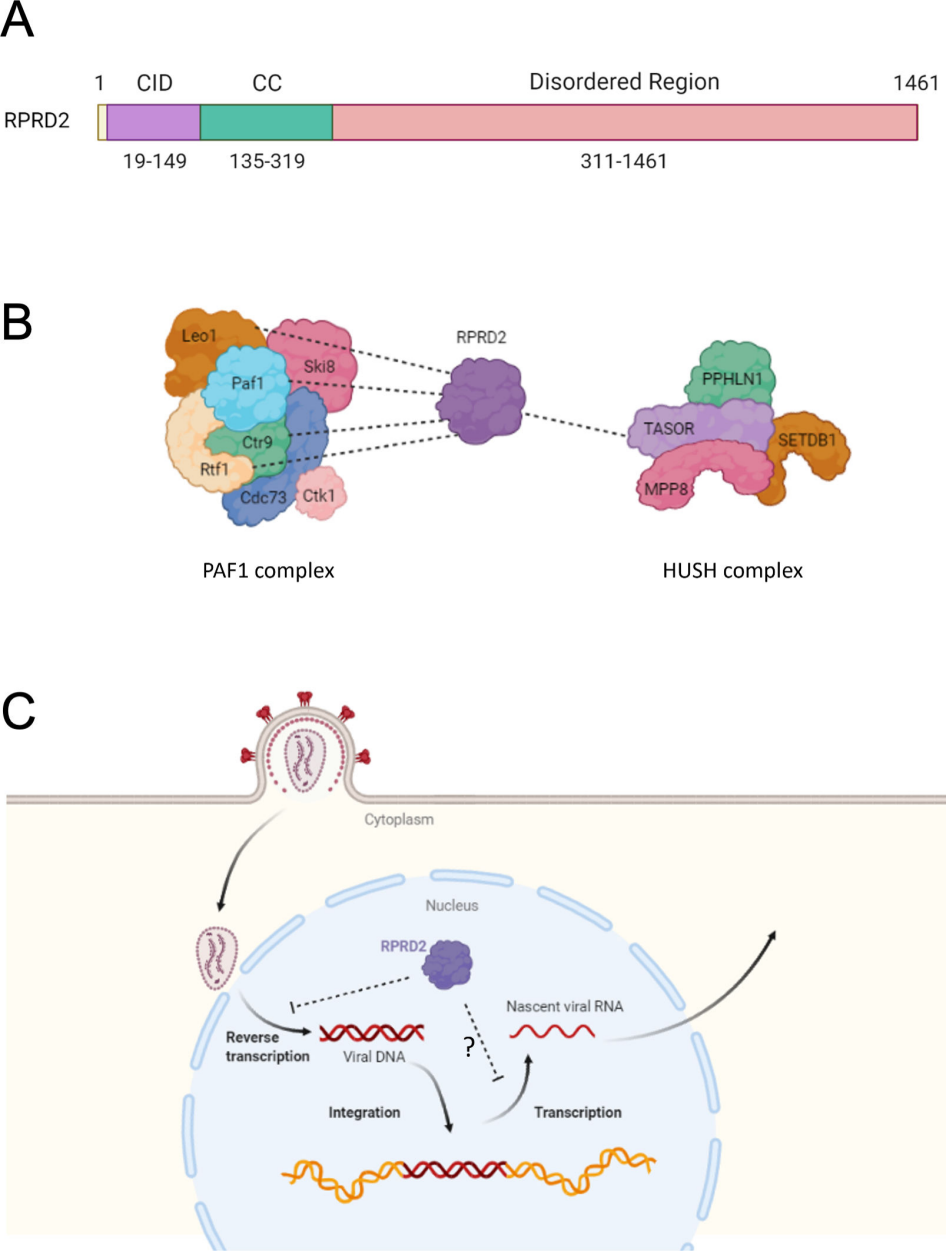
Characteristics of the anti-retroviral host protein RPRD2/RNA-associated early-stage antiviral factor. (A) Schematic representation of the size and domain structure of RPRD2 protein. Numbers represent predicted amino acid locations of each domain. CID, RNA polymerase II C-terminal-interacting domain; CC, coiled-coil domain. (B) Predicted and experimentally validated interactions between RPRD2 and members of the HUSH and PAF1 complexes are shown with dotted lines. (C) RPRD2 restricts HIV infection by inhibiting reverse transcription and potentially also nascent transcription of integrated provirus.

In addition to these protein complex members was the relatively uncharacterized protein; regulation of nuclear pre-mRNA domain containing 2 (RPRD2) (Fig. 2A). RPRD2 is constitutively expressed and mainly found within the nucleus (63). The N-terminal is structurally similar to the proteins RPRD1A and RPRD1B which have roles in the cell cycle, and all three possess predicted coiled-coil domains and RNA polymerase II C-terminal-interacting domains (CID), allowing them to interact with the C-terminal tail of RNA polymerase II (64). However, the RPRD2 polypeptide is approximately fivefold longer than RPRD1A or RPRD1B and predicted to have a large, disordered C-terminal domain containing many phosphorylation sites.

This screen identified several proteins that phenocopied the Lv2 restriction seen previously, many of which have roles in similar pathways. Regulation of nucleic acids seemed particularly important, with many hits governing reverse transcription, integration, and transcription. However, cellular proteins rarely have a single function so other effects of these proteins on the HIV replication cycle could not be ruled out.

### RNA-associated early-stage antiviral factor

By 2014, Marno et al. had begun to characterize RPRD2 and its anti-retroviral activity. Upon siRNA knockdown of RPRD2, a more than 50-fold increase in HIV-1 infection was seen, and overexpression of a GFP-RPRD2 fusion protein led to an inhibition of viral replication as measured by p24 immunostaining (65). Human RPRD2 was also shown to inhibit infection of HIV-2 and strains of simian immunodeficiency virus (SIV) from African green monkey, macaque, and sooty mangabey by between 15- and 126-fold, showing that RPRD2 is a generalized lentiviral restriction factor (65).

RPRD2 knockdown resulted in an increase of both early and late reverse transcripts within 2 hours of viral challenge, indicating that RPRD2 acts before or during the reverse transcription process (65). Conversely, when GFP-RPRD2 was overexpressed, fewer infected cells were detectable and fewer early and late reverse transcripts were produced by 8 hours post-infection (65). The effects of RPRD2 knockdown were more pronounced than its overexpression, perhaps suggesting that RPRD2 does not wholly account for the Lv2 restriction, and other components are to be involved. Nevertheless, these results suggested that RPRD2 acts early in infection and leads either directly or indirectly to a decrease in viral reverse transcripts.

RPRD2 protein was precipitated when poly(A) specific oligo(dT) was used as bait, indicating RPRD2 binds RNA with a poly(A) tail. However, treatment with DNase prevented this precipitation (65), suggesting that RPRD2 specifically interacts with complexes containing both RNA and DNA. Treatment with a mix of RNase A, which specifically cleaves ssRNA, and RNase H, which degrades DNA:RNA hybrid complexes, did not reduce RPRD2 association with poly(A) RNA. Indeed, the amount of RPRD2 precipitated was slightly increased at higher concentrations of these enzymes (65). This may be due to RPRD2 binding more tightly to the DNA component of DNA:RNA hybrids, such that additional binding sites for RPRD2 are revealed as more RNA is degraded. Due to the characteristics of nucleic acid binding and early-phase HIV restriction, RPRD2 was given a second name, RNA-associated early-stage antiviral factor (REAF). However, the authors stated that their experiments did not rule out an additional antiviral role for RPRD2 post-reverse transcription.

Interestingly, RPRD2 antiviral activity was found to be route of entry-dependent consistent with Lv2. RPRD2 knockdown by siRNA did not affect the infectivity of HIV-1(89.6)Δ*env* pseudotyped with VSV-G, whereas the same knockdown increased the infectivity of wild-type HIV-1(89.6) by approximately 10-fold (65). VSV-G triggers entry by clathrin-mediated endocytosis through a ubiquitously expressed glycolipid (24), suggesting RPRD2-mediated restriction of HIV-1(89.6) is circumvented by a VSV-G-mediated route of entry.

A key characteristic of restriction factors is the co-evolved ability of the virus to target and interfere with the host protein inhibiting it. For example, APOBEC3G is overcome by the HIV-1 viral infectivity factor (Vif) (9). Marno et al. (65) showed that RPRD2 protein is quickly decreased in response to HIV-1 infection, but recovers by 2 hours post-infection, although RPRD2 mRNA levels remain constant throughout infection. This degradation was blocked by treatment with the proteasome inhibitor MG132, indicating the protein is targeted to the conventional proteasome. The degradation was also blocked in the presence of the reverse transcriptase inhibitor azidothymidine (AZT), suggesting that initiation of reverse transcription is required to trigger this temporal degradation (65).

This study was crucial in defining RPRD2 as a host restriction factor by clearly showing its antiviral effect against HIV-1, HIV-2, and SIV, explaining its mechanism of blocking HIV infection by reducing the levels of reverse transcripts produced, and confirming it is counter-targeted by the incoming virus. Aligning with earlier work that indicated that the Lv2 restriction was route of entry dependent, RPRD2 restriction also depends on the entry mechanism, suggesting RPRD2 as the cellular determinant of the Lv2 phenotype, although the full mechanism is still not clear. The evidence that supports RPRD2 binding to DNA:RNA hybrids suggests that it is interfering with reverse transcription directly; however, this binding activity could also be explained by binding to DNA:RNA hybrids that are produced during DNA repair (66) or transcription (67).

### RPRD2 is a major component of Lv2 restriction

Since the antiviral activity of RPRD2 was discovered using an Lv2-sensitive virus (41) and found to be route of entry dependent (41, 65) similar to Lv2 restriction (23), RPRD2 seemed likely to be at least a partial component of Lv2 restriction. As outlined above, the Lv2 phenotype was originally characterized using two differentially sensitive HIV-2 molecular clones of isolates derived from the same patient, HIV-2 MCR and HIV-2 MCN (22, 23). Marno et al. used these molecular clones, in addition to specific site-directed mutants, to examine whether RPRD2 is partially or completely responsible for Lv2 restriction (68).

Consistent with RPRD2’s role as a potent inhibitor of retroviral infection, depleting RPRD2 led to a 50-fold increase in HIV-2 MCR infectivity (68), showing the restricted molecular clone is highly susceptible to RPRD2 activity. Loss of RPRD2 also led to a 10-fold increase in HIV-2 MCN infectivity compared to control (68), showing that even apparently resistant viruses still retain some susceptibility to RPRD2. To investigate whether RPRD2 restriction has the same capsid and envelope viral determinants as Lv2 (23, 33, 35), Marno et al. inserted both *env* and *gag* genes from Lv2-restricted HIV-2 MCR in place of HIV-2 MCN *env* and *gag* (68). This chimera virus was 66-fold restricted by RPRD2, whereas the reciprocal chimera virus was only restricted threefold (68). Additionally, RPRD2 was found to be highly expressed in cells previously shown to have a strong Lv2 activity, including PBMCs (23, 68), and have lower expression in cells lacking Lv2 restriction. From these results, the authors concluded that the antiviral mechanism of RPRD2 is consistent with Lv2 restriction.

Schmitz et al. previously mapped the Lv2 capsid protein determinant to a single amino acid mutation at capsid position 73 (position 207 in the Gag polyprotein) (23), which is within the binding domain of cleavage and polyadenylation-specific factor 6 (CPSF6) (69, 70). Introducing the I73V mutation to the Lv2-restricted HIV-2 MCR capsid protein resulted in a much lower increase in infectivity upon RPRD2 depletion than seen for wild-type HIV-2 MCR (68). Notably, this I73V mutation phenocopied the well-studied N74D mutation in terms of reduced ability to interact with truncated CPSF6 (CPSF6-358), and furthermore, N74D also increased susceptibility to RPRD2 restriction. This raises the question of whether CPSF6 trafficking of HIV capsid is involved in the route of entry-dependent restriction by Lv2 and RPRD2. Adding the equivalent HIV-2 capsid protein I73V mutation to HIV-1 also led to a more potent restriction by RPRD2 compared to wild-type HIV-1 NL4.3 capsid protein (68), identifying I73V as a determinant of susceptibility for HIV-1, too, although weaker compared to the phenotype seen in HIV-2. More potent determinants for RPRD2 restriction were identified within HIV-1 capsid at P38A, G89V, and G94D, further confirming that capsid protein is a viral determinant of susceptibility to Lv2/RPRD2 for HIV-1.

As with HIV-2, HIV-1 envelope was found to rescue Lv2/RPRD2-sensitive capsid proteins from restriction, confirming that the inhibition is route of entry dependent (Fig. 1) (68). However, the precise envelope determinants have not been mapped and the precise routes of permissive and restricted entry have not been elucidated. Compared to R5/dual-tropic HIV-1(89.6), the X4-tropic HIV-1 NL4.3 was significantly more resistant to RPRD2 restriction (68), suggesting that HIV tropism could play a role in whether the virus utilizes a restricted or permissive route of entry. It is known that some envelope proteins are more able to rescue restricted cores than others, for example, HIV-1 NL4.3 X4-tropic envelope is poor at rescue (23, 33, 68). Whether this is due to overcoming a restriction or using a more efficient entry pathway is as yet unclear. Regarding restriction, it is tempting to speculate on the involvement of IFITM proteins, which can influence envelope in a chemokine co-receptor and route of entry-dependent manner (39). However, although HIV-1 entry through endocytic pathways has been described, it remains a contentious topic (36, 37). Notably, a preprint including affinity proteomic analysis of RPRD2 revealed an interaction with APLN (apelin) (71) which inhibits HIV-1 entry to the cell (72) via its endogenous affinity for the G-protein-coupled apelin receptor which has been shown to act as an alternative co-receptor for HIV-1 and SIV under some experimental circumstances (73), though it is not clear how this could contribute toward the Lv2 phenotype. Nonetheless, a better understanding of context-dependent HIV entry pathways might illuminate understanding of the envelope-dependent component of Lv2.

Overall, using the original restricted (MCR) and non-restricted (MCN) HIV-2 molecular clones (22, 23) confirmed the suggestions of earlier work that RPRD2 and Lv2 are mechanistically similar. The effects of RPRD2 phenocopied Lv2 and have the same envelope and capsid viral determinants. Collectively, these findings suggest that RPRD2 is a major component of the Lv2 restriction. The next step was to further understand how RPRD2 and HIV interact on a molecular level.

### HIV-1 accessory protein Vpr interacts with RPRD2 in the nucleus and targets it for degradation

The HIV-1 accessory protein Viral protein R (Vpr) has been shown to alter the level of approximately 2,000 cellular proteins (74) and accordingly enhances viral replication in macrophages and, to a lesser extent, cycling T cells (75, 76). Vpr is packaged into the virion, released shortly after entry, and rapidly localizes to the nucleus (77), suggesting it is required in the early phase of infection.

Gibbons et al. showed that Vpr interacts with endogenous RPRD2 and targets it for degradation (63). This degradation was complete within 30 minutes of viral entry and occurred even when Vpr alone was delivered to primary macrophages, while HIV-1(89.6) bearing a *vpr* deletion [HIV-1(89.6)Δ*vpr*] was no longer capable of degrading RPRD2 (63). This depletion of RPRD2 was observable despite a cellular response to viral infection which increased expression levels of RPRD2 in both HeLa cells and monocyte-derived macrophages. Knockdown of RPRD2 using shRNA resulted in increased infectivity of wild-type HIV-1(89.6), but critically, a significantly greater increase of infectivity was seen for HIV-1(89.6)Δ*vpr* when RPRD2 was depleted (63), reinforcing that Vpr degrades RPRD2 to overcome its restrictive action.

Crucially, nuclear localization of Vpr and its ability to interact with cullin 4A-DBB1 (DCAF1) E3 ubiquitin ligase were shown to be requirements for RPRD2 degradation (63). Although at the time, the predominant model was that capsid core breakdown and reverse transcription occurred in the cytoplasm, several studies have more recently shown that intact capsid can traverse the nuclear pore and reverse transcription is not complete until after nuclear entry (78–84). Capsid breakdown and release of viral nucleic acid occurring inside the nucleus are consistent with the majority of RPRD2 localizing to the nucleus as RPRD2 has been shown to affect reverse transcription and bind reverse transcripts (65). There is, however, a small but significant proportion of RPRD2 in the cytoplasm, especially during interphase (63), raising the question of what, if any, is its cytoplasmic antiviral role if reverse transcription is occurring in the nucleus. Whether RPRD2 found in the cytoplasm could bind and saturate incoming Vpr before it translocates to the nucleus, thereby allowing the nuclear restriction activity of RPRD2 to go unhindered, is an interesting question. When HeLa-CD4 cells were infected with HIV-1(89.6)Δ*vpr*, there was a small increase in RPRD2 protein levels in the cytoplasm but a stronger increase in the nucleus which stayed high (63). Since new protein is synthesized in the cytoplasm, the lower cytoplasmic increase in RPRD2 upon depletion suggests rapid protein localization to the nucleus, pointing to the nucleus as the more important site of antiviral action. Interestingly, previous work with wild-type HIV-1(89.6) concluded that RPRD2 mRNA levels remained constant throughout infection (65), suggesting that the increase in RPRD2 protein in the absence of Vpr may be due to another accessory protein or perhaps another Vpr target stabilizing RPRD2 protein upon infection.

Interestingly, knockdown of RPRD2 has been shown to cause an accumulation of cells in G2/M (63, 71, 85), signifying cell cycle arrest, suggestive of a role in cell cycle progression for RPRD2. Furthermore, a preprint paper suggested that even a relatively small increase in RPRD2 constitutive expression negatively affected cell growth, and higher overexpression of RPRD2 almost completely abolished cell growth (71). This is evocative of the induction of G2/M arrest due to the expression of Vpr (74, 86, 87), although to a weaker extent, suggesting that Vpr-mediated degradation of other factors in addition to RPRD2 results in this phenotype. It is increasingly apparent that Vpr is relatively promiscuous in terms of its protein-protein interactions (74, 88), and likely functions at the pathway level to modulate transcription and innate immune function for the benefit of the virus. Knockdown of several other Vpr-targeted proteins also only weakly induces G2/M arrest (74), and furthermore, it has been suggested that the G2/M cell cycle arrest induced by Vpr is not the intended function of this accessory protein but actually a side effect of Vpr-targeted degradation of host factors involved in DNA repair (74, 89, 90). However, Vpr-mediated G2/M cell cycle arrest could provide a replicative advantage since it delays cells at that point in the cell cycle at which the HIV LTR is most active (91–93), and this arrest has been shown to prevent phosphorylation and activation of the HUSH complex member transcription activation suppressor (TASOR) (55). Zhang and Bieniasz identified coiled-coil domain containing 137 (CCDC137) as a target of Vpr that when depleted not only more closely mimicked the HIV-1 infection-triggered Vpr-mediated G2/M cell cycle arrest but also produced the enhanced HIV-1 gene expression (94), suggesting it is a core Vpr target. Overall, the function of Vpr and the link between cell cycle arrest and DNA damage induced by Vpr is still controversial (91, 95–97), and understanding the relationships and interactions between the many targets of Vpr may be revealing.

The discovery that HIV-1 targets RPRD2 for degradation via its accessory protein Vpr strengthens RPRD2 as a host viral restriction factor since the virus has evolved to block and counter-attack antiviral mechanisms. Potential future questions include whether RPRD2 binds incoming Vpr in the cytoplasm or the nucleus, and whether the increase in RPRD2 levels means less Vpr is available for binding other targets. Vpr has also been described to overcome the inhibition of transcription mediated by the HUSH complex (53, 61, 98) and the HUSH complex member TASOR also binds RPRD2 (99). Thus, it is a tempting proposition to consider that the HUSH-mediated silencing of HIV-1 and inhibition by RPRD2 are functionally linked, especially given the influence of RPRD2 on cellular transcription (71). However, it is not yet known if RPRD2 specifically affects HIV-1 transcription or latency as is seen with the HUSH complex (56). It is worth noting that in the original paper characterizing Lv2 restriction, the *vpr* gene was identical between MCR and MCN clones and so was not followed up in the gene swapping experiment. Although the MCR Vpr was likely functional against RPRD2, the small effect size was likely masked by the much larger effect of envelope and capsid. These original experiments were carried out in HIV-2 which also has Vpx. Whether HIV-2 Vpx can also overcome RPRD2-mediated restriction remains to be seen, although RPRD2 was not identified in a Vpx interaction screen (53, 98). Although Vpr targets RPRD2 for degradation, it is clearly not sufficient to fully overcome the restriction. Indeed the complete picture of RPRD2 activity may be as complex as the Lv2 phenotype itself. Evidence that RPRD2 appears necessary for normal cell survival and maintenance suggests it also has an important role beyond viral restriction, as is common for restriction factors; however, this had not been well studied until recently.

### RPRD2 negatively regulates global transcription

Antiviral restriction factors commonly have roles in cellular maintenance outside of infection, and their functionality is co-opted and adapted to respond to invading pathogens. The main focus of characterizing the RPRD family proteins has been RPRD1A and RPRD1B which affect cell cycle, Wnt signaling, carcinogenesis, and transcription through their RNA polymerase II CID (64, 100, 101). RPRD2 also has a CID (Fig. 2A) (64) placing it at the site of transcription and has been shown to bind RNA with several nucleic acid species (65, 102), indicating that RPRD2 could have a role in RNA processing and metabolism. Furthermore, the yeast RPRD1A/RPRD1B/RPRD2 homolog RTT103 has been shown to control transposition of the Ty1 retrotransposon (103), most likely via transcriptional termination (104, 105). Given this, and RPRD2’s nuclear localization, RPRD2 could be interacting with and regulating oligonucleotides that result from the nascent transcription of host, endogenous retroelement, or viral DNA, in addition to those produced by reverse transcription.

In a preprint paper characterizing RPRD proteins, RPRD2 precipitation co-purified multiple subunits of the RNA polymerase II complex (71), as predicted by RPRD2’s CID. RPRD2 binding to RNA polymerase II was maintained in high stringency conditions, indicating the high strength of this interaction; however, after 4 hours of treatment with the transcriptional inhibitor 5,6-dichloro-1-β-D-ribofuranosylbenzimidazole (DRB), the RPRD2-RNA polymerase II interaction was diminished (71), indicating that RPRD2 binds to actively transcribing RNA polymerase II. Other binding partners included proteins involved in immune processes, E3 ubiquitin ligase TRIM21, and heat shock proteins (71) considered critical for maintaining cellular proteostasis (106). Gene ontology (GO) analysis showed that, overall, proteins interacting with RPRD2 are involved in antiviral and stress responses, and immune pathways responding to viral infections (71).

Although the DNA:RNA hybrids formed during transcription are usually transient, R-loops consisting of a DNA:RNA hybrid and the associated non-template single-stranded DNA can be dangerous for genome stability (107). RPRD2 has been identified as a regulator of R-loops via a proximity-dependent labeling system TurboID which is described by the authors as able to identify R-loop binding proteins that bind the ssDNA component (108), which would agree with the previous suggestion that RPRD2 binds preferentially to the DNA strand in DNA:RNA hybrids (65).

Winczura et al. (71) show in their preprint that RPRD2 negatively regulates nascent human transcription genome-wide (71). Depletion of RPRD2 protein resulted in increased nascent RNA production of mRNA, long noncoding RNAs and small nuclear RNAs but not RNA polymerase I-transcribed rRNA. Overexpression led to downregulation of transcription across the same RNA subtypes (71). Knockdown of RPRD2 led to an increase in RNA polymerase II transcription rate, indicating that RPRD2 negatively regulates RNA polymerase II transcription rate which is the likely cause for the changed levels of nascent RNA (71).

The vast majority of genes whose transcription was affected by depletion of RPRD2 had unaffected steady-state levels (71), presumably due to compensatory mechanisms of the cell to adjust RNA levels by inhibiting or enhancing RNA degradation pathways. For the 64 genes that were differentially transcribed and also altered at the steady-state level upon RPRD2 depletion, the most significant GO terms associated with these genes were transcriptional pathways, followed by those involved in immunological responses (71), suggesting RPRD2 could be acting specifically to upregulate a program of antiviral immune response genes. Of note, both the PAF1 and the HUSH complexes have recently been identified as having roles in regulating the immune response. By interacting with specific chromatin regions and potentially recruiting sequence-specific transcription factors, the PAF1 complex promotes the expression of genes related to the antiviral, antimicrobial, and inflammatory responses (109–111), whereas by regulating long interspersed element 1 (LINE-1), the HUSH complex prevents the induction of interferon-stimulated genes via sensing of double-stranded DNA and JAK/STAT signaling (112). Regulation of the immune response might be consistent with the high expression of RPRD2 seen in myeloid cells (63).

RPRD2 has also been shown to interact with members of the PAF1 complex (113) and the HUSH complex (99), both of which were identified in the same screen for HIV-1 restriction factors as RPRD2 (41) and have also been identified as regulating host and viral transcription (53, 61, 62, 98, 114). While investigating protein-binding targets of the HUSH complex, Douse et al. identified RPRD2 as a strongly enriched interactor of the HUSH component TASOR, even above the two other recognized members of the HUSH complex, M-phase phosphoprotein 8 (MPP8) and perphilin (PPHLN1) (99). In support of joint functionality, RPRD2, TASOR, and other members of the HUSH complex were identified in a screen for inhibitors of LINE-1 retroelement mobility, in which TASOR and other HUSH components were shown to specifically inhibit retroelement transcription (115). Furthermore, both RPRD2 and TASOR are degraded by both HIV-1 Vpr (74). Separate from HUSH, RPRD2 also co-purifies with the PAF1 complex and 1-β-D-ribofuranoside (DRB) sensitivity-inducing factor (44, 113), which can regulate transcription both negatively and positively by associating with RNA polymerase II, for example, it can interact with the negative elongation factor to promote stalling of RNA polymerase II at some genes (116).

A better understanding of the relationship between RPRD2, with its newly described role in transcription, and potential relationship with the HUSH and PAF1 complexes would aid understanding of HIV infection as well as gene regulation. Notably, expression of the PAF1 complex has been linked to HIV-1 elite controllers and long-term non-progressors (117), and PAF1 complex member CTR9 was found to have increased expression in HIV-DNA+ primary memory T cells compared to HIV-DNA- memory T cells from the same patients (118). Whether RPRD2, which appears to be functionally linked to PAF1, influences this phenomenon is not yet known. However, the described enrichment of function regarding innate immune genes suggests studying RPRD2-driven gene expression in the context of innate immune cells, particularly during viral infection or cell stress may be of interest for further study.

## DISCUSSION

Since the first description of the Lv2 phenotype in 2001, substantial progress has been made in understanding this restriction of HIV infection. The cellular factor RPRD2 seems to drive the majority of the phenotype but another factor(s) may also be involved. It is now understood that the restriction occurs at reverse transcription and that viral *gag*, *env,* and *vpr* all drive viral susceptibility. A range of lentiviruses including HIV-1, HIV-2, and SIV are affected by Lv2 and RPRD2 restriction. Despite this progress, there are still substantial gaps in our understanding of Lv2/RPRD2 function.

### Lv2 restriction and HIV-1 entry

Perhaps the greatest deficit in understanding the Lv2 phenotype surrounds virus entry. HIV-1 bearing a susceptible core and entering via the cell surface can evade Lv2 restriction while virus entering through endosomes is restricted. This leads to a proposal that HIV-1 entering the cell through different routes faces different fates. This would require analysis of post-entry using imaging-based approaches, though the enthusiasm for such work would be difficult when there is a lack of consensus about whether HIV can genuinely yield productive infection via endosomes. Furthermore, the data regarding VSV-G pseudotypes are confounding, as VSV-G enters via endosomes but is resistant to Lv2 restriction, unlike what is seen for HIV envelopes that use endocytic entry, suggesting there may be some nuance that runs counter to an oversimplified endocytic hypothesis.

The roles of cellular factors in the entry component of Lv2 restriction are also not fully understood. AP-2 (34, 41) and dynamin-2 (41), both involved in endocytosis, were identified as contributing to the Lv2 phenotype, but whether any inhibitory factors further feed into Lv2/RPRD2 restriction and patterns of viral susceptibility is not yet clear. Presumably, such factors may be present in the initial screen which found RPRD2; this may require sub-screens performed in the context of RPRD2 modulation.

Whether any parallels can be made regarding the route of entry and virus fate in the context of other restrictions is worth considering. Restriction outcome can be altered through the use of VSV-G pseudotyping in the unmapped Lv3 restriction seen in macaque cells (28), though examples of entry pathways affecting intracellular restriction are not common. The most beneficial field of study in regard to better understanding the entry contribution of Lv2/RPRD2 restriction may then be a better definition of post-entry steps of HIV-1, an area already of immense interest and subject to recent changes in understanding (78–84). How these new studies can reframe pre-existing data and interpretation should be considered.

### RPRD2 and reverse transcription

RPRD2 has been shown to inhibit reverse transcription, but the precise mechanism is still unclear. RPRD2 may bind nucleic acids during the process of reverse transcription and in doing so either block the process sterically or through recruitment of other factors. Potentially, RPRD2 is repressing reverse transcriptase in a similar manner to its repression of RNA polymerase II (71).

Various studies have identified RPRD2 as binding to RNA with a poly(A) tail (65), DNA:RNA hybrids (65, 107), as well as late-stage reverse transcripts which consist of double-stranded DNA (65). Given the presence of DNA:RNA hybrids in both reverse transcription and nascent transcription, it is feasible that regulators that recognize or are recruited to these hybrids in cellular transcription could also regulate reverse transcription. Binding to all three nucleic acid species has been shown for other proteins that regulate transcription (119–121), some of which bind both RNA and DNA at the same domain while others are capable of binding RNA and DNA simultaneously. It has been suggested that these promiscuous binding domains may have less stringent criteria for interacting with nucleic acids or may modify their structure when DNA and RNA compete for the same interaction surface (121). Whether RPRD2 is capable of binding RNA and DNA at the same time and with the same or distinct domain is yet to be determined. It is worth noting that large, disordered regions, such as the RPRD2 C-terminal, are commonly involved in nucleic acid binding (122). It is interesting to note that with the addition of DNase, but not RNase, the binding of RPRD2 to DNA:RNA hybrids was lost (65), suggesting that RPRD2 is binding in a DNA-dependent manner to these molecules.

### RPRD2 and transcriptional regulation

RPRD2 also represses global transcription in uninfected human cells by slowing RNA polymerase II (71), although the mechanism is unknown. It seems likely that it is directly affecting elongation through its interaction with the C-terminal tail of RNA polymerase II and the nascent RNA protruding from the active center of the enzyme. It may be that RPRD2 drives transcriptional termination like its yeast homolog (103–105). Whether it works with the HUSH and/or PAF1 complexes or is functionally independent of them remains to be seen. One possibility is that RPRD2 recruits the PAF1 complex to drive proximal pausing at HUSH loci.

A host restriction factor affecting multiple steps of the HIV-1 replication cycle is not unprecedented. TRIM28 inhibits HIV-1 integration by recruiting histone deacetylase 1 (HDAC1) to deacetylate HIV-1 integrase (123) and also represses transcription of endogenous retroviruses (124, 125) and integrated murine leukemia virus (126–128). More recently, TRIM28 has been shown to promote HIV-1 latency by SUMOylating CDK9 and inhibiting positive transcription elongation factor b (P-TEFb) (14). Similarly, SAM and HD domain containing deoxynucleoside triphosphate triphosphohydrolase 1 (SAMHD1) blocks reverse transcription (13, 129) and was initially suggested by some to degrade viral RNA (130), although this was disproved by other studies (131). Recently however, SAMHD1 was shown to function as an ssRNA 3´ exonuclease and degrade total RNA, poly(A)-mRNA, and ssRNA oligonucleotides with a 5´-cap or 5´-triphosphate (132). Furthermore, depletion of several members of the PAF1 complex was shown to increase the number of both early and late HIV-1 reverse transcription products in addition to the abundance of proviral DNA (41). The PAF1 complex is also required for histone H3 methylation on lysines 4 and 79 by COMPASS methyltransferase leading to gene silencing in yeast (45) and increased heterochromatin stability (133).

It was notable that the vast majority of genes whose transcription was affected by depletion of RPRD2 had unaffected steady-state levels (71). The cell has many mechanisms to buffer mRNA levels including by inhibiting or enhancing RNA degradation pathways. Some preliminary evidence suggests that RPRD2 may interact with XRN1 and XRN2 (134, 135), RNA exonucleases that degrade RNA in the nucleus. Potentially, RPRD2 could be recruiting RNA degradation factors to degrade viral nucleic acids during reverse transcription and/or transcription, especially in light of the increasingly important role exonucleases and endonucleases are being assigned in control of transcription (136–141). The HUSH complex has recently been described as recruiting the RNA decay nuclear exosome targeting complex to transposable element transcription sites to degrade nascent RNA (142), in addition to repressing transcription via histone methylation. Given its ability to bind mRNA, RPRD2 could also be directly targeting nucleic acid species for degradation in a similar manner or indeed mediating RNA decay either through its interaction with the HUSH complex or independently.

Reverse transcription of HIV-1, integration of the proviral genome, and nascent viral transcription have recently been shown to occur in a spatially and temporally closer manner than previously thought (78–80, 82, 84, 143). These processes are all likely occurring in nuclear speckles (82, 144), liquid-liquid phase separated foci within the nucleus. Proteins with large, disordered regions (or low complexity[LC] domains), such as RPRD2, are enriched in liquid-liquid phase separation and are commonly RNA-binding proteins (122, 145). These data together suggest that RPRD2 is at the site of HIV-1 transcription and can interact with the transcription machinery to potentially negatively regulate nascent transcription of HIV-1 provirus in addition to affecting reverse transcription. Although RPRD2 was identified as a repressor of LINE-1 retroelement mobility (115), it was not explored whether it was specifically inhibiting retroelement transcription or the reverse transcription step, and though Winczura et al. identified transcription of many species of RNA as affected by RPRD2 (71), they did not describe the effect of RPRD2 on endogenous retroelements.

Whether RPRD2 affects retroviral gene expression with any specificity is not yet known. If confirmed, it might be possible to consider RPRD2 in the context of latency reversal, currently a central component of HIV cure strategies. Indeed, even if RPRD2 does not affect transcription of integrated HIV then, it raises the question of how HIV DNA differs from host chromatin and the similarities between regulation of host and viral transcription. Clarification of the binding activity and affinity of RPRD2 for different nucleic acid species would also help unravel the protein’s role in transcription processes, for example, is the preference context or location specific?

### Potential role for RPRD2 in innate immunity

The Lv2 restriction was first defined in monocyte-derived macrophages (22) and it was subsequently shown that RPRD2 knockdown enhanced HIV-1 production in such cells. RPRD2 was also found to be very highly expressed in the nucleus of both M1 and M2 polarized macrophages, as well as dendritic cells (63). HIV-1 Vpr, which targets RPRD2 (among many other proteins), increases infectivity of HIV-1 to a greater extent in macrophages than in T cells (75, 76) and is known to influence nuclear factor kappa light chain enhancer of activated B cells (NFkB) activity in a context-dependent manner (63, 146). This raises the question of whether RPRD2 has a more fundamental role in innate immune antiviral responses and HIV control. However, RPRD2 is not under positive selection nor is it interferon-inducible (63). At present, it is unknown to what extent RPRD2 influences innate immune gene expression in myeloid cells; however, transcripts increased in expression upon RPRD2 knockdown were enriched in innate immune genes in HEK293T cells (71). PAF1, which interacts with RPRD2 (113), has been linked to the expression of interferon-stimulated genes during influenza A infection (109–111). Therefore, it would be interesting to study whether RPRD2 can influence antiviral gene signatures.

### Conclusion

The Lv2 restriction phenotype is predominantly driven by RPRD2, but questions remain over other restriction factors and route of entry. It seems plausible that Lv2 is not a singular restriction but a complex combination of factors in part driven by viral entry route and trafficking. Whether the PAF1 complex and HUSH complexes identified in the same screen that found RPRD2 also contribute to the phenotype remains to be seen. RPRD2 is a potent HIV restriction factor but its precise mechanism of action is still yet to be uncovered. Binding of nucleic acid by RPRD2 seems likely to be important for its function in both inhibiting reverse transcription and nascent transcription, but may also be linked to induction of innate immune antiviral responses. The extent to which RPRD2 cooperates with other factors to affect HIV transcription remains open. Further elucidation of RPRD2 mechanisms will benefit our understanding of innate immune control and pathogenesis of HIV-1 infection but may also have wider implications for the regulation of cellular transcription.
